# Therapeutic relevance of an EU-GMP certified *Cannabis sativa* L. strain in a dual *in vivo* model of cognitive impairment and chronic neuropathic pain

**DOI:** 10.3389/fphar.2026.1761426

**Published:** 2026-02-20

**Authors:** Ivona Costachescu, Raluca-Maria Gogu, Gabriela-Dumitrita Stanciu, Carmen Solcan, Loredana Horodincu, Andrei Szilagyi, Vlad-Constantin Craciun, Daniela-Carmen Ababei, Bogdan-Ionel Tamba

**Affiliations:** 1 Grigore T. Popa University of Medicine and Pharmacy Iasi, Advanced Research and Development Center for Experimental Medicine “Prof. Ostin C. Mungiu” - CEMEX, Iasi, Romania; 2 Faculty of Veterinary Medicine, “Ion Ionescu de la Brad” University of Life Sciences, Iasi, Romania; 3 Department of Computer Science, “Alexandru Ioan Cuza” University of Iasi, Iasi, Romania; 4 Grigore T. Popa University of Medicine and Pharmacy Iasi, Pharmacodynamics and Clinical Pharmacy Department, Iasi, Romania; 5 Grigore T. Popa University of Medicine and Pharmacy Iasi, Department of Pharmacology, Clinical Pharmacology and Algesiology, Iasi, Romania

**Keywords:** Alzheimer’s disease, Cannabis sativa L., chronic intermittent therapy, comorbidities, EU-GMP certification, shared neurobiological mechanisms

## Abstract

**Background:**

Alzheimer’s disease (AD) is a progressive neurodegenerative disorder characterized by cognitive decline and frequently co-occurs with chronic pain. Worldwide, over 55 million people are affected by AD, with nearly half experiencing persistent pain. Chronic pain has been linked to accelerated memory deterioration and an increased risk of dementia, but the interplay between these conditions remains poorly understood. Existing therapies for AD and chronic pain are limited in efficacy, highlighting the need for interventions targeting multiple pathological pathways. The endocannabinoid system, which is altered in both AD and chronic pain, represents a potential therapeutic target, though its role in AD patients with comorbid pain remains unexplored.

**Methods:**

The study evaluated the effects of an EU-GMP certified *Cannabis sativa* L. strain (5 mg/kg, Cannabixir® Medium Flos) on neurobiological alterations in a rat model designed to explore mechanistic interactions between scopolamine-induced transient cognitive impairment and chronic neuropathic pain induced by unilateral sciatic nerve ligation. Treatment outcomes were assessed through nociceptive tests, clinical monitoring and tissue analyses to examine cognitive and pain-related effects.

**Results:**

Cannabixir® Medium Flos induced robust, time-dependent analgesia in thermal nociceptive tests, with the combination of the *Cannabis sativa* L. strain, donepezil and tramadol producing significantly longer response latencies than tramadol alone. Mechanical sensitivity was minimally affected across treatments. Immunohistochemical analyses revealed that Cannabixir® Medium Flos, either alone or in combination with donepezil or tramadol, produced the most pronounced neuroprotective effects, reducing astrocytic (GFAP) and microglial (Iba1) activation, lowering Caspase-3 and IL-6 expression, and preserving both hippocampal neuronal integrity as well as peripheral nerve structure.

**Conclusion:**

These findings indicate that Cannabixir® Medium Flos, particularly when combined with donepezil and tramadol, provides superior analgesic and neuroprotective effects compared to tramadol alone. Its multi-target action - alleviating thermal nociception, reducing neuroinflammation, limiting apoptosis and preserving neuronal and peripheral nerve integrity—supports its potential as an adjunct therapy in managing dementia with comorbid chronic neuropathic pain. Future studies should explore the molecular mechanisms underlying these effects and assess long-term safety and efficacy across diverse models of neurodegeneration and chronic pain.

## 1 Introduction

Alzheimer’s disease (AD) is the most common neurodegenerative disorder worldwide, affecting over 55 million people, with projections estimating nearly 139 million cases by 2050 (Alzheimer’s Association, 2025). AD is characterized by progressive neuronal loss, synaptic dysfunction and cognitive decline, which lead to substantial functional impairment and dependence (Blanton et al., 2023). Alongside cognitive deficits, chronic pain is a frequent but underrecognized comorbidity in AD, although the reported prevalence varies substantially across studies due to differences in assessment tools, disease stage and data sources (van Kooten et al., 2016; Yao et al., 2025). According to the International Association for the Study of Pain (IASP), pain is defined as an unpleasant sensory and emotional experience associated with or resembling, actual or potential tissue damage (Raja et al., 2020). Chronic pain, lasting 3 months or longer, affects over 30% of the global population and is particularly prevalent among older adults (Cohen et al., 2021). In individuals with AD, pain is often expressed non-verbally through behaviors such as frowning, groaning or agitation, and its intensity may fluctuate across disease stages (Liao et al., 2021; Khalid et al., 2022).

Chronic pain exacerbates behavioral and neuropsychiatric symptoms and has been associated with accelerated cognitive decline, whereas neurodegenerative processes linked to cognitive impairment may, in turn, amplify pain perception (Palesi et al., 2016; Atee et al., 2021; Guerreiro et al., 2022). Consistently, patients with chronic pain are reported to be 2.2 times more likely to develop cognitive impairment than those without pain (Huai et al., 2021; Kumaradev et al., 2021). Moreover, a broad spectrum of pain phenotypes has been described in patients with dementia, including musculoskeletal, osteoarticular and headache-related pain (Lin et al., 2018); however, neuropathic pain remains comparatively less systematically investigated and may be underestimated due to communication difficulties and atypical behavioral manifestations in cognitively impaired individuals (Yao et al., 2025). This clinical complexity highlights the need for translational experimental models capable of interrogating shared pathological pathways between chronic pain and neurodegeneration (Niziński et al., 2026). Taken together, these observations provide the rationale for investigating neuroinflammatory and neurodegenerative mechanisms in preclinical models, rather than attempting to fully reproduce the complex clinical phenotype of Alzheimer’s disease.

The mechanisms underlying the interplay between AD and chronic pain are complex and multifactorial. Overactivation of microglia and neuroinflammation, brain structure changes and disruptions in neural circuits contribute both to neuropathic pain and to the progression of AD pathology (Landrø et al., 2013; Samartin-Veiga et al., 2019). Persistent pain triggers neuroinflammatory cascades mediated by activated glial cells, which in turn exacerbate neuronal excitability, increase pain sensitivity and impair cognitive function (Ji et al., 2018; Vergne-Salle and Bertin, 2021). Among the molecular systems implicated, the endocannabinoid system (ECS) - comprising cannabinoid receptors type 1 and 2 (CB1 and CB2), endogenous ligands and metabolizing enzymes - is significantly altered in both chronic pain and AD (Kanwal et al., 2024).

ECS is widely distributed along pain-processing pathways within the central and peripheral nervous systems, as well as in neuroimmune circuits (Finn et al., 2021). Experimental evidence reveals that cannabinoids exert analgesic actions in a variety of preclinical models of acute and chronic pain of diverse etiologies (Wilson et al., 2008; Cox et al., 2007). These effects appear to result from modulation of nociceptive transmission at multiple levels (peripheral, spinal and supraspinal), either by attenuating ascending pain signals or by enhancing descending inhibitory control (Cichewicz and McCarthy, 2003; Cox et al., 2007). At the peripheral level, cannabinoids have been shown to suppress nociceptive input when administered locally, an effect mediated through both CB1 and CB2 receptors (Potenzieri et al., 2008). Within the brain, activation of CB1 receptors by agonists such as Δ^9^-tetrahydrocannabinol engages descending inhibitory pathways involving key pain-modulatory centers (Calignano et al., 1998). In addition to the direct modulation of pain signaling, cannabinoids may also exert analgesic effects indirectly through their anti-inflammatory properties. CB1 and CB2 receptor activation, as well as stimulation of other cannabinoid-sensitive targets, contributes to the suppression of pro-inflammatory cascades (DeLong et al., 2010; Wen et al., 2023). Selective CB2 receptor agonists have demonstrated analgesic efficacy in experimental models of inflammatory and neuropathic pain by reducing inflammatory signaling (Malan Jr et al., 2003; Wen et al., 2023).

Despite accumulating evidence on the interplay between cognitive decline and chronic pain, no previous studies, to our knowledge, have systematically evaluated standardized, EU-GMP certified *Cannabis sativa* L. strains in preclinical models designed to investigate mechanistic interactions between peripheral nerve injury and transient cholinergic dysfunction. The strain employed in this study possesses a well-characterized cannabinoid profile and EU-GMP certification ensures reproducibility and compliance with stringent European pharmaceutical standards. Furthermore, our research group has established its safety through comprehensive acute and chronic toxicology studies, providing a rigorous preclinical basis for further evaluation (Filipiuc et al., 2023; Stanciu et al., 2024; Tudorancea et al., 2025).

In the present study, scopolamine was employed to induce transient cholinergic dysfunction and cognitive impairment, a widely used preclinical model for investigating mechanisms underlying memory deficits (Stanciu et al., 2023; Miravalles et al., 2025). Donepezil was included as a reference cognitive drug; it primarily acts through cholinesterase inhibition, but also exerts neuroprotective and anti-inflammatory effects, supporting its use as a comparator in preclinical models of cognitive deficits (Sheikh and Ammar, 2024). Tramadol served as a standard analgesic to validate the pain model; although it is less commonly used for neuropathic conditions and has known central nervous system effects, observational evidence indicates that its use is not associated with an increased risk of Alzheimer’s disease (Oh et al., 2024), justifying its inclusion as a pharmacological benchmark in this preclinical context, while acknowledging that anti-epileptics or antidepressants are more typical first-line choices for neuropathic pain in elderly populations.

## 2 Materials and methods

### 2.1 Animal care

Naturally aged male Sprague-Dawley rats (14 months old, n = 56) obtained from the Cantacuzino Institute, Romania, were utilized. The animals were housed in individually ventilated cages (IVCs) in pairs throughout the study within the animal facility of the Advanced Research and Development Center for Experimental Medicine, “Prof. Ostin C. Mungiu” - CEMEX. Standard husbandry conditions were maintained throughout the study, including a temperature of 20 °C ± 4 °C, relative humidity of 50% ± 5%, and a 12-h light/dark cycle. Food and water were available *ad libitum*. All experimental procedures were performed at CEMEX following the European Union Directive 2010/63/EU and Romanian Law No. 43/2014 regarding the ethical use of animals in research. The study protocols received prior approval from both the Ethical Committee of Grigore T. Popa University of Medicine and Pharmacy, Iaşi (approval no. 519/25.01.2025) and the Romanian National Sanitary Veterinary and Food Safety Authority (approval no. 76/24.02.2025).

### 2.2 Reagents and study design

The *Cannabis sativa* L. phytocomplex used in the present study (Cannabixir® Medium Flos, PZN: 7001905; Cansativa GmbH, Mörfelden-Walldorf, Germany) was certified as compliant with EU-GMP quality standards. According to the manufacturer’s Certificate of Analysis (No. POO5840/11.05.2022), the product contained 15.6% Δ^9^-tetrahydrocannabinol (THC) and less than 1% cannabidiol (CBD). The strain of the medicinal product was manufactured in line with the principles of EU Good Manufacturing Practice and the European Pharmacopoeia, following the 2017 guidance issued by the German Federal Institute for Drugs and Medical Devices. Analytical testing was performed in a licensed facility authorized under Section 13 of the German Medicinal Products Act (Arzneimittelgesetz, AMG).

Scopolamine hydrobromide (≥98% purity) was obtained from Sigma-Aldrich (St. Louis, MO, USA). Donepezil was obtained as Aricept® 10 mg orodispersible tablets (Pfizer, New York, USA) and tramadol as 100 mg orodispersible tablets (KRKA, Bucharest, Romania). Isoflurane (inhalation vapour, liquid) was purchased from Laboratorios Karizoo, S.A. (Caldes de Montbui, Spain). From the Epredia Holdings Ltd. (Portsmouth, NH, USA) the following was purchased: ready-to-use decalcifying solution, xylene and paraffin infiltration medium (Histoplast PE). Formalin was supplied by VWR Chemicals (Radnor, PA, USA), and ethanol (99.9%) by Laboratorium Life Science (Galați, Romania). Mayer’s hematoxylin, eosin Y, and hematoxylin for counterstaining were purchased from Sigma-Aldrich (Burlington, USA). The Masson-Goldner Trichrome staining kit was obtained from Morphisto GmbH (Offenbach am Main, Germany). All compounds were stored and handled as described in the product information sheets.

The dried cannabis inflorescences (Cannabixir® Medium Flos, PZN: 7001905) were mechanically ground to a fine powder using an RM 200 mortar grinder (Retsch GmbH, Haan, Germany), then passed through a 125 μm mesh sieve (BSS No. 120). The resulting homogeneous powder was suspended in a 0.1% sodium carboxymethylcellulose (CMC-Na) solution and administered by oral gavage at a dosing volume of 0.5 mL/100 g body weight. Scopolamine was dissolved in physiological saline (0.9% NaCl), whereas donepezil and tramadol tablets were powdered and suspended in a 0.1% CMC-Na solution. Each compound was administered in a separate gavage to ensure accurate dosing and avoid potential interactions during administration. Dosage selection was based on previously validated experimental models of Alzheimer’s disease and interspecies dose conversion factors. The final administered doses were: donepezil 0.65 mg/kg (clinically equivalent) (Zhang et al., 2019), scopolamine 3 mg/kg (Fatima et al., 2017; Thongrong et al., 2024) and tramadol 10 mg/kg (Pandita et al., 2003; Lavasani et al., 2013). The dose of Cannabixir® Medium Flos (5 mg/kg) was determined based on prior pharmacokinetic studies and safety data from our group, ensuring a pharmacologically relevant exposure well below reported toxic thresholds (Filipiuc et al., 2023).

### 2.3 Experimental design and treatment protocol

Following a 14-day acclimatization period, during which the rats were gently handled to reduce stress and familiarize them with the laboratory environment, anesthesia was induced to perform partial sciatic nerve ligation as a model of unilateral peripheral mononeuropathy. Induction was achieved using 3.5%–4% isoflurane in 95% oxygen and anesthesia was maintained at 1.5%–2% isoflurane in 95% oxygen throughout the procedure. Under anesthesia and sterile conditions, the left sciatic nerve was exposed at mid-thigh level. A single tight ligature was applied to approximately one-third to one-half of the nerve trunk just proximal to its trifurcation, following the method described by Nakamura et al. (2013). The incision was closed in layers and the animals were allowed 7 days to recover before further procedures. Sham-operated controls underwent the same surgical exposure of the left sciatic nerve without ligation, followed by closure of the incision. The contralateral (right) limb remained untouched in all animals. During the postoperative period, animals were monitored daily for mobility, wound healing and signs of distress. Analgesics were not administered to avoid interfering with the development of neuropathic pain behaviors.

After surgical recovery, rats were randomly assigned to eight experimental groups (n = 7 per group) and received treatments in repeated cycles of five consecutive days followed by 2 days of rest, for a total duration of 30 days. This regimen simulates chronic exposure while minimizing tolerance and stress (Mouro et al., 2018). The experimental groups are summarized in Table 1.

**TABLE 1:** Experimental groups and treatment regimens.

Group (n = 7)	Surgery	Drugs regime	Treatment period
ConG	Sham	0.1% CMC-Na (gavage)	days 1–30
SG	Sciatic nerve ligation	scopolamine: 3 mg/kg, ip	days 22–30
DG	Sciatic nerve ligation	donepezil: 0.65 mg/kg, gavage scopolamine: 3 mg/kg, ip	donepezil: days 1–30 scopolamine: days 22–30
CanG	Sciatic nerve ligation	Cannabixir®: 5 mg/kg, gavage scopolamine: 3 mg/kg, ip	Cannabixir®: days 1–30 scopolamine: days 22–30
TG	Sciatic nerve ligation	tramadol: 10 mg/kg, gavage scopolamine: 3 mg/kg, ip	Cannabixir®: days 1–30 scopolamine: days 22–30
CanDG	Sciatic nerve ligation	Cannabixir®, donepezil: Gavage scopolamine: ip	Cannabixir®, donepezil: days 1–30 scopolamine: days 22–30
CanTG	Sciatic nerve ligation	Cannabixir®, tramadol: Gavage scopolamine: ip	Cannabixir®, tramadol: days 1–30 scopolamine: days 22–30
CanDTG	Sciatic nerve ligation	Cannabixir®, donepezil, tramadol: Gavage, scopolamine: ip	Cannabixir®, donepezil, tramadol: days 1–30, scopolamine: days 22–30

Scopolamine was administered during the last 9 days to induce a pharmacological model of cognitive impairment. Donepezil served as a reference treatment for cholinergic modulation and tramadol was used as a standard analgesic for neuropathic pain.

### 2.4 Pain-related behavioral tests

Nociceptive thresholds were evaluated on day 7 after surgery and at the end of the experimental period (week 4). A decrease of at least 20% in the nociceptive threshold compared with baseline values and the sham-operated control group for any of the applied stimuli was considered indicative of neuropathy development (Le Bars et al., 2001). Behavioral testing was performed immediately before compound administration and subsequently at 30, 60, 90 and 120 min to assess time-dependent effects. The battery of behavioral tests included the Randall–Selitto test (mechanical nociception; Ugo Basile analgesimeter, model 37,215), Tail Flick test (thermal nociception; Ugo Basile apparatus, model 7360/05,998) and Hot Plate test (thermal pain sensitivity; Ugo Basile apparatus, model 7280). All procedures were conducted in accordance with the methodology previously described by our team (Filipiuc et al., 2024), under standardized conditions of handling, habituation and measurement to minimize stress and interindividual variability. While our study focused on nociceptive outcomes, cognitive performance was not assessed in this experimental setting. Previous work from our group demonstrated that oral administration of the same EU-GMP-certified *Cannabis sativa* L. strain (15.6% THC) at comparable or higher doses (2.5–10 mg/kg, up to 600 mg/kg) does not produce measurable central effects (Filipiuc et al., 2023). The absence of behavioral or cognitive alterations in these chronic studies can be explained by the pharmacokinetic profile of the compound: psychoactive THC remains undetectable in serum and is largely replaced by tetrahydrocannabinolic acid (THCA-A), which has markedly reduced blood–brain barrier permeability, minimal CB1 receptor activity and primarily mediates peripheral effects via transient receptor potential (TRP) channels and cyclooxygenase-1/cyclooxygenase-1 (COX1/COX-2) interaction. Its short plasma half-life (T_1_/_2_ ≈ 5.3 h) and rapid systemic clearance preclude significant accumulation, supporting that the lack of central behavioral changes.

### 2.5 Sample collection and biochemistry analysis

At the end of the experimental period, animals were anesthetized with isoflurane and blood was collected by intracardiac puncture into 5 mL clot activator tubes for subsequent biochemical analysis. Following blood collection, animals were euthanized by an overdose of isoflurane. Blood samples were allowed to clot at room temperature for 30 min and then centrifuged at 1,500 *g* for 15 min at 4 °C to obtain serum. The evaluated markers included alanine aminotransferase (ALT), aspartate aminotransferase (AST), creatinine (CRE), total cholesterol (CHOL), glucose (GLU), albumin (ALB), alkaline phosphatase (ALP), total protein (TP), urea (UREA) and total bile acids (TBIL). These biochemical measurements were performed to monitor hepatic, renal and metabolic function, thereby assessing the systemic safety profile of the *Cannabis sativa* L. treatment and reference drugs (donepezil and tramadol). Calibration and quality control procedures were performed according to standard laboratory protocols.

### 2.6 Histological assessment and immunohistochemical analysis

A complete necropsy was performed on each animal, including both external inspection and internal examination to identify any macroscopic alterations. Key organs—including the brain, ligated sciatic nerve, and spinal cord (particularly the lumbar region) — were harvested to assess neuropathic and neurodegenerative changes. Additional organs, such as the liver, kidneys, heart, lungs, gastrointestinal tract (small and large intestines) and spleen, were collected to monitor systemic safety and detect potential off-target effects of the administered treatments. Harvested tissues were fixed in 10% formalin (prepared from 36% stock solution) for 24–48 h. The spinal cord was decalcified for 7 days in a ready-to-use decalcifying solution prior to processing.

Tissues were trimmed along organ-specific anatomical planes and processed following established protocols (Magaki et al., 2019; Guerin, 2023; Stanciu et al., 2024) using the Excelsior™ AS Tissue Processor through a standardized sequence: dehydration in graded ethanol, clearing in xylene and infiltration with paraffin. Samples were then embedded in paraffin blocks and sectioned at 3–4 μm thickness using a semi-automatic microtome (CUT 5062, SLEE Medical GmbH, Nieder-Olm, Germany). Sections were stained with hematoxylin and eosin (H&E) using Mayer’s hematoxylin and 0.5% aqueous eosin Y. The sciatic nerve was additionally stained with the Masson-Goldner Trichrome kit.

Immunohistochemical analysis was performed to investigate cellular and molecular alterations associated with neuropathic pain and neuroinflammatory processes, and to evaluate the potential neuroprotective and anti-inflammatory effects of the tested compounds. Specifically, markers of neuronal integrity (NeuN), astrocytic activation (glial fibrillary acidic protein - GFAP), microglial activation (ionized calcium-binding adapter molecule 1 - Iba-1), apoptosis (Caspase-3) and inflammation (interleukin-6 - IL-6, hemoglobin–haptoglobin scavenger receptor - CD-163) were assessed in the spinal cord, sciatic nerve and selected brain regions. Sections were deparaffinized in xylene, progressively rehydrated through graded ethanol to distilled water, and subjected to antigen retrieval in 10 mM citrate buffer (pH 6) using a microwave oven (10 min, 95 °C). Slides were allowed to cool for 20 min at room temperature and rinsed twice in phosphate-buffered saline (PBS, 5 min each). Endogenous peroxidase activity was blocked with 3% hydrogen peroxide, followed by two PBS washes. Sections were incubated overnight at 4 °C with primary antibodies (Table 2) in a humid chamber. After three PBS washes, secondary antibodies were applied using IgG detection (Novolink Polymer Detection Systems, Leica Biosystems, Goettingen, Germany). Immunoreactivity was visualized with 3,3′-diaminobenzidine (DAB) and counterstained with hematoxylin. Prepared slides were examined under a Leica DM750 light microscope at ×400 magnification. Representative images were digitally captured and analyzed by a board-certified veterinary histopathologist. Image analysis was performed using QuPath software (version 0.5.1) at ×50 magnification. Images were processed with the brightfield H-DAB IHC module, calibrated from pixels to micrometers, and regions of interest were annotated. Marker-specific scoring compartments were defined (NeuN–nucleus; GFAP/Iba-1 – cell; Caspase-3/IL-6/CD-163 – cytoplasm) with threshold 1+ set at 0.3. Positive cells were expressed per µm^2^ of regions of interest (ROI). Data are presented as means ± standard deviation (SD).

**TABLE 2:** The primary and secondary antibodies used for immunohistochemistry, including their specific dilutions.

Primary antibody	Dilution	Secondary antibody	Dilution
Anti-Iba-1 antibody (10904-1-AP), Proteintech (Manchester, United Kingdom)	1:1,000	Goat anti-rabbit IgG H&L (HRP) (ab205718) Abcam (Cambridge, United Kingdom)	1:1,000
Anti-interleukin-6 antibody (XG360436), Invitrogen (Massachusetts, USA)	1:100	Goat anti-mouse IgG H&L (HRP) (ab97023) Abcam (Cambridge, United Kingdom)	1:100
Anti-GFAP antibody (173,002), SYSY antibodies (Goettingen, Germany)	1:1,000	Goat anti-rabbit IgG H&L (HRP) (ab205718) Abcam (Cambridge, United Kingdom)	1:1,000
Anti-CD163 antibody (MA5-16658), Invitrogen (Massachusetts, USA)	1:100	Goat anti-mouse IgG H&L (HRP) (ab97023) Abcam (Cambridge, United Kingdom)	1:100
Anti-Caspase3 antibody (43-7800), Invitrogen (Massachusetts, USA)	1:100	Goat anti-mouse IgG H&L (HRP) (ab97023) Abcam (Cambridge, United Kingdom)	1:100
Anti-Neu N antibody (26975-1-AP), Proteintech (Manchester, United Kingdom)	1:1,000	Goat anti-rabbit IgG H&L (HRP) (ab205718) Abcam (Cambridge, United Kingdom)	1:1,000

Iba-1: ionized calcium-binding adapter molecule 1; GFAP: glial fibrillary acidic protein; CD163: hemoglobin–haptoglobin scavenger receptor; NeuN: neuronal nuclei; HRP: horseradish peroxidase; IgG: immunoglobulin G.

### 2.7 Statistical analysis

Statistical analyses were performed using Python 3.11.9 with the pandas, matplotlib, bioinfokit, scipy, and statsmodels libraries. For body weight analysis, the data were visualized in two ways: weight trends based on mean values of individuals within each group, and differences in group means compared with the control group (ConG, reference). For the Tail-Flick, Randall–Selitto and Hot-Plate tests, a one-way ANOVA was computed across all groups for each selected time point. Since all three experiments shared the same six variables (post-operatory, T0, T30, T60, T90 and T120), each figure includes six subplots displaying both theoretical quantile plots and boxplots for all eight groups (ConG, SG, DG, TG, CanG, CanDG, CanTG and CanDTG). Based on the high degree of similarity among group biochemical data, Pearson’s correlation was applied between the mean values of each experimental group and those of the control group (ConG, reference).

Quantitative immunohistochemical data are reported as means ± standard deviation (SD). Differences between experimental and control groups were analyzed using one-way analysis of variance (ANOVA) with Tukey’s *post hoc* test for multiple comparisons. A p-value <0.05 was considered statistically significant.

## 3 Results

### 3.1 General health status

The effects of Cannabixir® Medium Flos (5 mg/kg) administration on body weight were monitored throughout the experimental period, with animals weighed once weekly, every Monday. Changes were expressed as percentage variation relative to baseline values recorded before treatment start. Statistical analysis revealed no significant differences in weekly body weight between treated and control groups. Absolute distances between group means showed that most groups (ConG, SG, DG, CanG, CanDG, CanTG, CanDTG) followed a similar increasing trend, whereas the TG group exhibited a slight decreasing trend. Importantly, this deviation in the TG rats did not reach statistical significance, indicating that treatment did not adversely affect overall body mass or general health status.

Serum biochemical parameters were evaluated after 30 days of treatment with Cannabixir® Medium Flos (5 mg/kg) or vehicle. As shown in Table 3, no statistically significant differences were observed between the cannabinoid-treated and control groups across any of the analyzed markers, including alanine aminotransferase (ALT), aspartate aminotransferase (AST), creatinine (CRE), total cholesterol (CHOL), glucose (GLU), albumin (ALB), alkaline phosphatase (ALP), total protein (TP), urea (UREA) and total bile acids (TBIL). Pearson correlation analysis further supported these observations by comparing the biochemical profiles of experimental groups with the reference group (ConG). Most groups displayed very high correlation coefficients (r > 0.98), confirming that the overall serum biochemical patterns remained largely consistent with the reference. A slightly lower correlation (r = 0.94) was observed for the TG group, reflecting minor physiological variability that did not reach statistical significance and was consistent with the mild deviations in weight trends noted for this group. These findings indicate that the compound did not induce systemic biochemical alterations, suggesting good tolerability under the experimental conditions.

**TABLE 3:** Serum biochemical profile of rats following a 30-day administration of Cannabixir® Medium Flos (5 mg/kg).

Biochemical parameter	ConG	SG	DG	TG	CanG	CanDG	CanTG	CanDTG
CRE (mean value)	0.65	0.65	0.73	0.71	0.59	0.7	0.69	0.71
AST (mean value)	70.17	83.51	75.12	67.51	80.75	53.45	52.8	63.94
ALT (mean value)	38.52	37.57	38.68	40.65	66.52	35.21	38.35	44.73
CHOL (mean value)	95.42	88.28	101.28	89.71	98.65	85.42	75.66	92.83
GLU (mean value)	148.42	144.14	130.14	133.71	140.42	135.14	144.16	151
ALB (mean value)	27.55	23.41	23.59	23.58	28.47	29.014	22.93	26.79
ALP (mean value)	79.57	82.28	64.71	63.57	91	70.42	64.33	70.33
TP (mean value)	55.64	51.91	55.97	56.78	54.6	54.87	52.81	53.23
UREA (mean value)	30.82	29.48	28.69	29.04	51.41	47.82	28.75	48.66
TBIL (mean value)	55.14	75.28	72.42	90.71	84.28	38	51.33	63.83
Pearson-correlation	​	0.978	0.971	0.94	0.951	0.973	0.985	0.982

ALT: alanine aminotransferase; AST: aspartate aminotransferase; CRE: creatinine; CHOL: total cholesterol; GLU: glucose; ALB: albumin; ALP: alkaline phosphatase; TP: total protein; UREA: urea; TBIL: total bile acids; ConG: control group - sham-operated rats; SG: scopolamine group; DG: donepezil group; CanG: Cannabixir® Medium Flos group; TG: tramadol group; CanDG: Cannabixir® Medium Flos + donepezil group; CanTG: Cannabixir® + tramadol group; CanDTG: Cannabixir® + donepezil + tramadol group.

### 3.2 The antinociceptive potential of Cannabixir® Medium Flos

A total of seven rats per experimental group (n = 7, Table 1) were included in all behavioral assessments. The battery of tests comprised the Tail Flick test, the Hot Plate test and the Randall–Selitto test, which were used to evaluate thermal and mechanical nociception at multiple time points over the treatment period.

**FIGURE 1:**
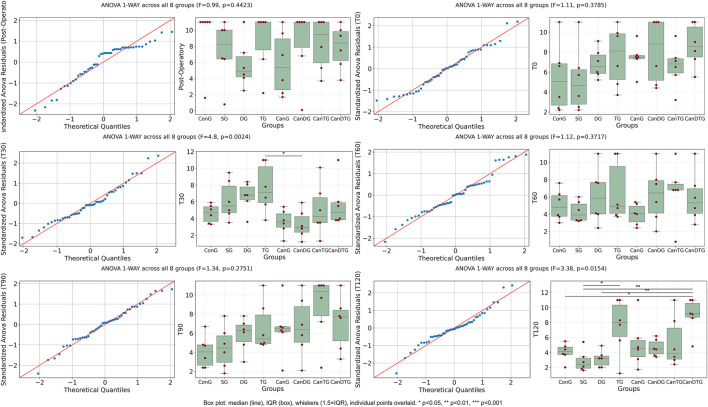
The impact of Cannabixir® Medium Flos on Tail Flick response. Tail Flick latencies were measured at six time points: post-operatory, T0, T30, T60, T90, and T120. Box plots show the median and interquartile range (IQR), with whiskers extending to 1.5×IQR and individual data points overlaid. One-way ANOVA was used to compare response times between treatment groups at each time point. While most time points showed no statistically significant differences, significant increases in latency were observed at T30 and T120 (p = 0.002 and p = 0.015, respectively), indicating a pronounced analgesic effect of Cannabixir® Medium Flos. ConG: control group - sham-operated rats; SG: scopolamine group; DG: donepezil group; CanG: Cannabixir® Medium Flos group; TG: tramadol group; CanDG: Cannabixir® Medium Flos + donepezil group; CanTG: Cannabixir® + tramadol group; CanDTG: Cannabixir® + donepezil + tramadol group.

Tail Flick response times were evaluated at six time points: post-operatory, T0, T30, T60, T90 and T120 (Figure 1). One-way ANOVA was used to compare values between groups at each time point. For most time points, no significant differences were observed between groups (p > 0.05). However, at T30 and T120, significant differences were detected (p < 0.05). Notably, at T120, all groups treated with the cannabinoid compound exhibited an increase in response time compared to the control groups, indicating an analgesic effect. The most pronounced effect was observed in the CanDTG group, which displayed the longest latency, even exceeding that of the tramadol-treated group. These results suggest that the cannabinoid compound produces a time-dependent analgesic effect, with the strongest response observed 120 min post-therapy.

**FIGURE 2:**
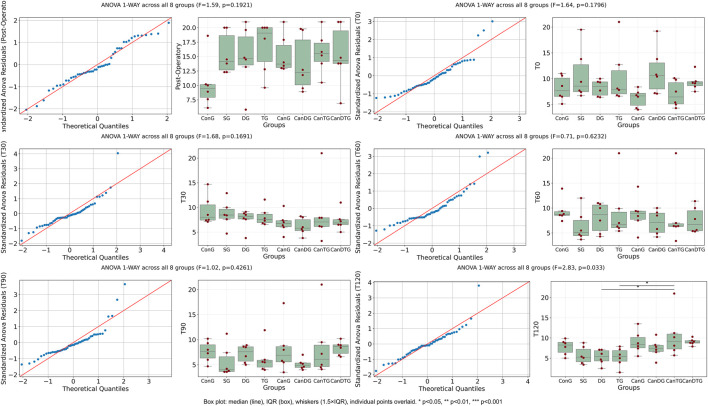
The effect of Cannabixir® Medium Flos on Hot Plate response. Response latencies were measured at six time points. Box plots show median and IQR with individual data points overlaid. One-way ANOVA was used to compare response times between treatment groups at each time point. No significant differences were observed at most time points (p > 0.05), with the exception of T120 (p = 0.033), where all cannabinoid-treated groups showed increased latency compared to control. ConG: control group - sham-operated rats; SG: scopolamine group; DG: donepezil group; CanG: Cannabixir® Medium Flos group; TG: tramadol group; CanDG: Cannabixir® Medium Flos + donepezil group; CanTG: Cannabixir® + tramadol group; CanDTG: Cannabixir® + donepezil + tramadol group.

Response latencies in the Hot Plate test were evaluated at six time points: post-operatory, T0, T30, T60, T90 and T120 (Figure 2). One-way ANOVA was used to compare response times between groups at each time point. For most time points, no statistically significant differences were observed (p > 0.05). The only significant difference was detected at T120 (p = 0.033). At this time point, all groups treated with the cannabinoid compound showed an increased response latency compared to controls, following the same trend observed in the Tail Flick test. This suggests that the cannabinoid compound produces a time-dependent analgesic effect, which becomes significant at 120 min post-treatment.

**FIGURE 3:**
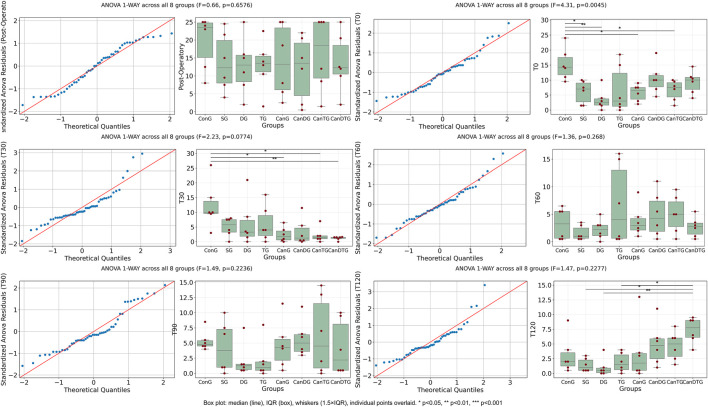
Mechanical nociceptive thresholds measured by the Randall-Selitto test after chronic intermittent oral Cannabixir® Medium Flos exposure in rats. Paw withdrawal thresholds were assessed at post-operatory, T0, T30, T60, T90 and T120. Box plots show median and IQR with individual data points overlaid. One-way ANOVA revealed a significant decrease in thresholds at T0 compared to Post-Operatory (p = 0.005), indicating successful induction of neuropathy. No significant differences were observed at later time points. ConG: control group - sham-operated rats; SG: scopolamine group; DG: donepezil group; CanG: Cannabixir® Medium Flos group; TG: tramadol group; CanDG: Cannabixir® Medium Flos + donepezil group; CanTG: Cannabixir® + tramadol group; CanDTG: Cannabixir® + donepezil + tramadol group.

Mechanical nociceptive thresholds were assessed using the Randall-Selitto test (Figure 3). A significant decrease in mechanical thresholds was observed at T0 compared to the post-operatory time point (p < 0.05), indicating that sciatic nerve ligation successfully induced neuropathy, establishing a baseline of mechanical hypersensitivity. At T30, the comparison approached significance but did not reach the threshold (p > 0.05), while later time points, including T90 and T120, showed similar non-significant differences (p = 0.224 and p = 0.228, respectively). These results suggest that the neuropathic pain model was effectively established, and no additional significant changes were observed in mechanical sensitivity over the course of the tested time points following treatment.

### 3.3 Impact of Cannabixir® therapy on neuroinflammatory and neuroregenerative processes in a dual *in vivo* model of cognitive impairment and chronic neuropathic pain

To further investigate the potential neuroprotective and neuroreparative effects of Cannabixir® therapy, histological and immunohistochemical analyses were performed to assess key markers associated with neuroinflammation, nerve regeneration, neuronal integrity and glial activation in a dual *in vivo* model combining chronic neuropathic pain and cognitive impairment.

**FIGURE 4:**
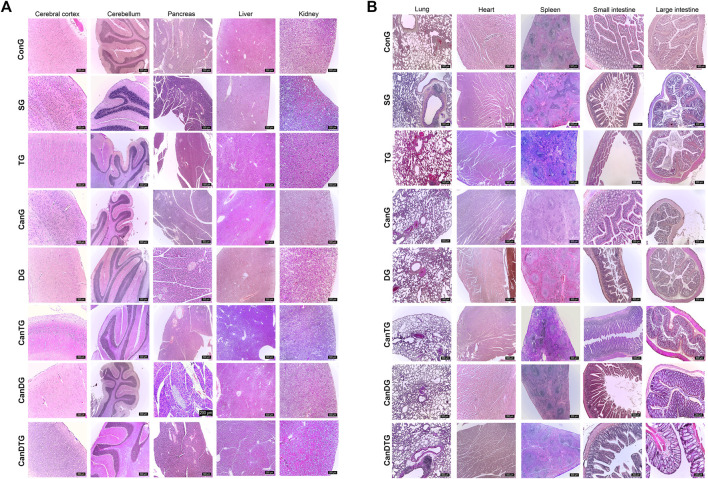
Histopathological evaluation of cannabinoid effects on internal organs. Chronic, intermittent treatment with Cannabixir® Medium Flos produced no significant histological alterations in haematoxylin–eosin–stained (H&E). **(A)** Sections of the cerebral cortex, cerebellum, pancreas, liver, kidneys. **(B)** Sections of the lung, myocardium, spleen, small and large intestine across the study groups, except in the TG group, where hepatic alterations were detected. H&E stain, scale bar 500 µm. ConG: control group - sham-operated rats; SG: scopolamine group; DG: donepezil group; CanG: Cannabixir® Medium Flos group; TG: tramadol group; CanDG: Cannabixir® Medium Flos + donepezil group; CanTG: Cannabixir® + tramadol group; CanDTG: Cannabixir® + donepezil + tramadol group.

Histological examination of the cerebral cortex, cerebellum and pancreas revealed no significant alterations in the studied groups, with the exception of the tramadol-treated group (TG). In this group, neurons in specific cortical regions appeared smaller, displaying densely condensed nuclei rich in heterochromatin—morphological features characteristic of apoptotic processes. In contrast, examination of peripheral organs showed localized hepatic alterations in the TG animals. Small focal areas of degeneration and oedema were identified in the liver, while in the CanTG group, these hepatic changes persisted, with oedema still being evident (Figures 4A,B).

**FIGURE 5:**
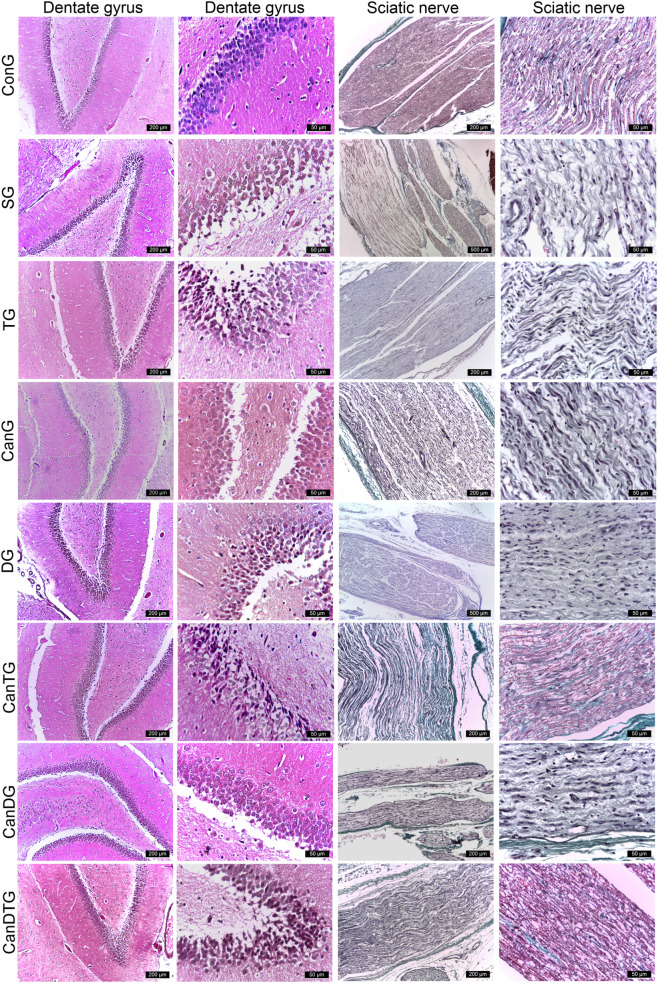
Histopathological evaluation of the post-ligation regions of the sciatic nerve and the dentate gyrus of the hippocampus. Masson’s trichrome and H&E stain, scale bar 50, 200 µm. ConG: control group - sham-operated rats; SG: scopolamine group; DG: donepezil group; CanG: Cannabixir® Medium Flos group; TG: tramadol group; CanDG: Cannabixir® Medium Flos + donepezil group; CanTG: Cannabixir® + tramadol group; CanDTG: Cannabixir® + donepezil + tramadol group.

Examination of the dentate gyrus within the hippocampus across all study groups revealed the presence of subgranular oedema in all animals. Rats exposed to scopolamine, tramadol or tramadol-containing combinations displayed a higher number of cells with densely condensed nuclei, accompanied by marked pericellular oedema (Figure 5).

Regarding the sciatic nerve, the post-ligation region exhibited normal morphology in the ConG rats, whereas degenerative changes were evident in the experimental groups. These alterations were less pronounced in the DG, CanG, CanDG, CanTG and CanDTG groups (Figure 5).

To evaluate the effects of the treatments on apoptosis, inflammation, immune cell infiltration and nerve regeneration in this *in vivo* dual model, immunohistochemistry (IHC) was performed using a panel of markers. Caspase-3 (Casp-3) was employed to assess neuronal apoptosis, while interleukin-6 (IL-6) served as an indicator of inflammatory signaling. CD163, a haemoglobin–haptoglobin scavenger receptor, was used to identify M2-polarized macrophages involved in anti-inflammatory and tissue repair processes. Iba1 (Ionized calcium-binding adapter molecule 1) marked microglia and macrophages, reflecting immune cell activation and infiltration. GFAP (glial fibrillary acidic protein) was used to assess Schwann cell and astrocytic activation, closely linked to glial reactivity and nerve regeneration. In addition, hippocampal immunohistochemistry was performed to evaluate the influence of drug therapy on scopolamine-induced cognitive impairment, using the same markers (GFAP, Casp-3, Iba1 and IL-6), complemented by NeuN (Neuronal Nuclei) as a specific marker of neuronal integrity and survival.

#### 3.3.1 Sciatic nerve findings

**FIGURE 6:**
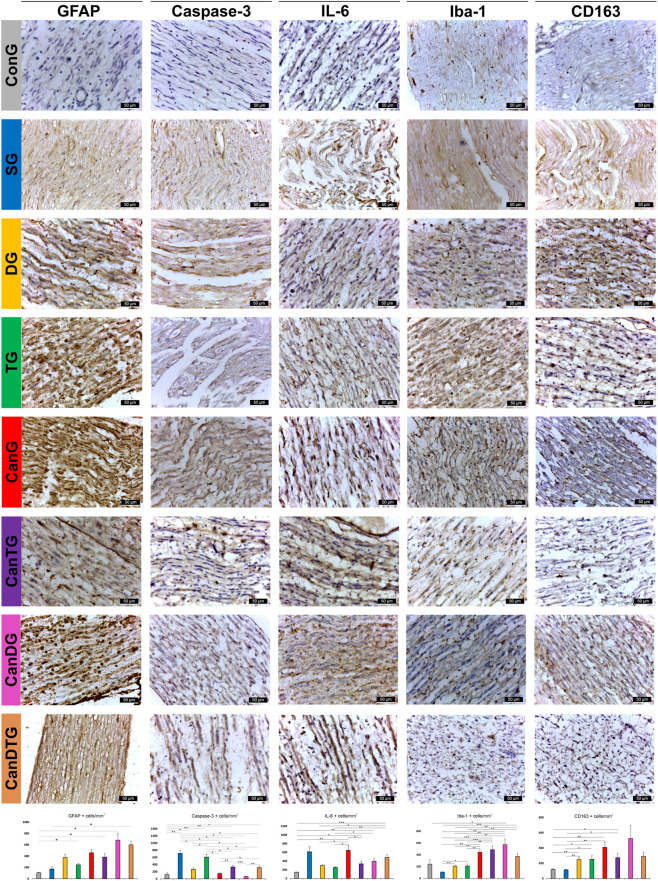
Representative immunohistochemical images of the sciatic nerve. Sections were stained for GFAP (astrocytic/Schwann cell activation), Caspase-3 (neuronal apoptosis), Iba1 (microglia/macrophage activation) and IL-6 (inflammatory signaling) across the experimental groups. Data are presented as means ± standard deviation (SD). Statistical analyses were performed using one-way ANOVA followed by Tukey’s *post hoc* test for multiple comparisons. Differences were considered statistically significant at p < 0.05. Scale bars = 50 μm. ConG: control group - sham-operated rats; SG: scopolamine group; DG: donepezil group; CanG: Cannabixir® Medium Flos group; TG: tramadol group; CanDG: Cannabixir® Medium Flos + donepezil group; CanTG: Cannabixir® + tramadol group; CanDTG: Cannabixir® + donepezil + tramadol group.

Thirty days post-sciatic nerve ligation, typical signs of axonal degeneration were observed, including myelinolysis, myelin debris and axonal fragmentation. Numerous regenerating axons were also identified: some forming clusters, others aligned in a 1:1 ratio with GFAP-positive Schwann cells, and a few exhibiting thin myelin sheaths indicative of remyelination. GFAP immunoreactivity in the control group was low (104.33 ± 5.95 positive cells/mm^2^). Compared with controls, tramadol treatment increased GFAP-positive cells by 136.40% (p < 0.05), while CanTG and CanDG groups showed more pronounced increases of 266.73% (p < 0.05) and 553.09% (p < 0.05), respectively. The SG displayed the lowest GFAP-positive cell density among all treatment groups (172.10 ± 35.24 cells/mm^2^), significantly different only from the CanDTG animals (p < 0.05). No significant differences were observed between CanDG and CanDTG groups (p = 0.585) (Figure 6).

Iba1-positive cells were identified across all experimental groups. The scopolamine group had the lowest density (105.69 ± 6.22 cells/mm^2^). Treatment with donepezil and tramadol significantly increased Iba1 immunoreactivity by 95.07% (p < 0.01) and 98.43% (p < 0.05), respectively. Inclusion of Cannabixir® Medium Flos, either alone or in combination with donepezil, tramadol or both, further increased Iba1-positive cell density (p < 0.01) compared with scopolamine or groups treated with donepezil or tramadol alone (Figure 6).

IL-6 immunoreactivity was significantly elevated in animals treated with tramadol (p < 0.001), Cannabixir® Medium Flos (p < 0.01), Cannabixir® Medium Flos–tramadol (p < 0.05), and Cannabixir® Medium Flos–donepezil–tramadol (p < 0.001) compared with controls. In nerve fibres, areas of oedema and degenerative changes were noted. Notably, combined Cannabixir® Medium Flos–tramadol treatment reduced IL-6 expression by 48.27% (p < 0.05) compared with cannabinoid alone (Figure 6).

Caspase-3 immunoreactivity was highest in the scopolamine (712.91 ± 80.71) and tramadol (607.82 ± 70.52) groups, with evident oedema and degenerative profiles in nerve fibres. Donepezil and Cannabixir® Medium Flos treatments significantly reduced Casp-3 expression compared with tramadol (p < 0.05), and Cannabixir® Medium Flos markedly decreased apoptosis by 75.64% relative to Cannabixir® Medium Flos–tramadol (p < 0.05). The lowest Casp-3 levels were observed in the Cannabixir® Medium Flos–donepezil group (62.26 ± 14.44), representing an 81.22% reduction versus CanTG (p < 0.001) and a 403.99% reduction compared with CanDTG (p < 0.01) (Figure 6).

CD163-positive macrophages were lowest in control (118.89 ± 13.26) and scopolamine (114.91 ± 6.18) groups. Significant increases were observed in DG (109.66%, p < 0.01), TG (111.42%, p < 0.05), CanG (242.95%, p < 0.01) and CanTG (130.04%, p < 0.05) groups. Among Cannabixir® Medium Flos -containing groups, no significant differences were found (Figure 6). Assessment of sciatic nerve fibre density 30 days post-ligation indicated that Cannabixir® Medium Flos therapy yielded the highest neuronal density (0.51 ± 0.01), significantly higher than all other groups (p < 0.001), followed by donepezil (0.051 ± 0.002), Cannabixir® Medium Flos–tramadol (0.044 ± 0.002), Cannabixir® Medium Flos–donepezil–tramadol (0.043 ± 0.001), Cannabixir® Medium Flos–donepezil (0.042 ± 0.003), tramadol (0.041 ± 0.002), scopolamine (0.037 ± 0.007) and control (0.039 ± 0.002) (Table 4).

**TABLE 4:** Neuronal density in the dentate gyrus and nerve fibers at 30 days after sciatic nerve ligation. Results are presented as means ± SD. Within each column, values with different superscript letters indicate a significant difference a, b, c, d, e, f, g, h — (p ≤ 0.05). A, B, C, D, E, F, G, H — (p ≤ 0.001).

Animal group	Density of neurons in the dentate gyrus (1 × 10^4^ cells/mm^2^)	Nerve fibre density (1 × 10^4^ fibre/mm^2^)
ConG group	0.26 ± 0.02^f^	0.039 ± 0.002^G^
SG group	0.13 ± 0.03^h^	0.037 ± 0.007^H^
DG group	0.36 ± 0.09 ^de^	0.051 ± 0.002^B^
TG group	0.23 ± 0.02^g^	0.041 ± 0.002^F^
CanG group	0.51 ± 0.08^a^	0.051 ± 0.013^A^
CanTG group	0.37 ± 0.07^d^	0.044 ± 0.002^C^
CanDG group	0.42 ± 0.03^c^	0.042 ± 0.003^E^
CanDTG group	0.48 ± 0.08^b^	0.043 ± 0.001^D^

ConG: control group - sham-operated rats; SG: scopolamine group; DG: donepezil group; CanG: Cannabixir® Medium Flos group; TG: tramadol group; CanDG: Cannabixir® Medium Flos + donepezil group; CanTG: Cannabixir® + tramadol group; CanDTG: Cannabixir® + donepezil + tramadol group.

#### 3.3.2 Hippocampal findings

**FIGURE 7:**
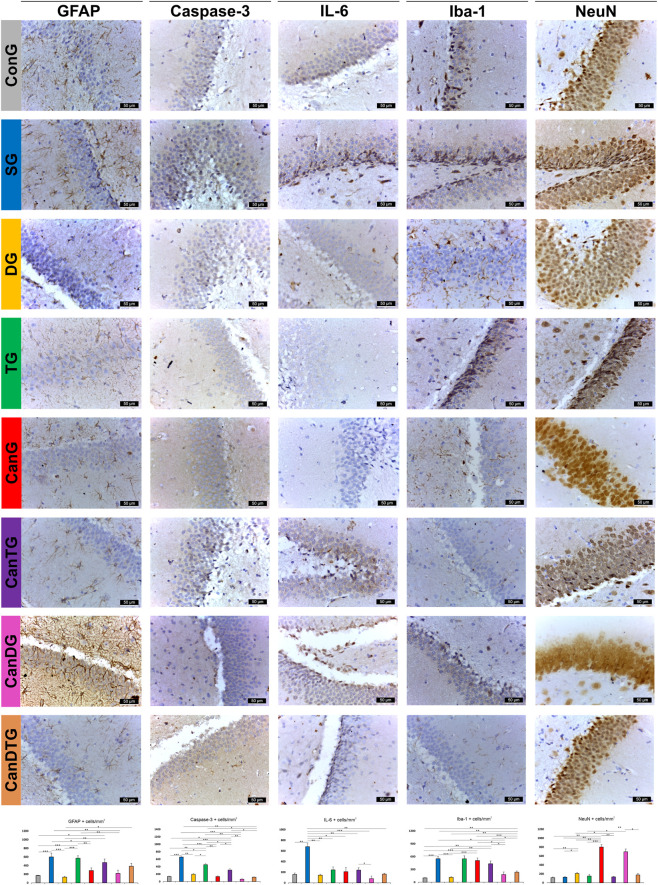
Representative immunohistochemical images of the hippocampus. Sections of the dentate gyrus were stained for GFAP (astrocytic activation), Caspase-3 (neuronal apoptosis), Iba1 (microglia activation), IL-6 (inflammatory signaling) and NeuN (neuronal integrity). Data are presented as means ± standard deviation (SD). Statistical analyses were performed using one-way ANOVA followed by Tukey’s *post hoc* test for multiple comparisons. Differences were considered statistically significant at p < 0.05. Images illustrate the different experimental groups, including ConG: control group - sham-operated rats; SG: scopolamine group; DG: donepezil group; CanG: Cannabixir® Medium Flos group; TG: tramadol group; CanDG: Cannabixir® Medium Flos + donepezil group; CanTG: Cannabixir® + tramadol group; CanDTG: Cannabixir® + donepezil + tramadol group. Scale bars = 50 μm.

In the hippocampus, GFAP-positive astrocytes were most numerous in the SG (600.43 ± 87.17) and TG (570.27 ± 58.51) groups, with no significant difference between them. Donepezil treatment significantly reduced astrocytic density (133.59 ± 18.82; 77.90% lower than scopolamine, p < 0.001). Cannabixir® Medium Flos addition also reduced GFAP immunoreactivity, with the lowest density observed in CanDG rats (221 ± 70.33), significantly lower than TG and SG groups (p < 0.01) (Figure 7).

In the dentate gyrus, glial cells displayed normal morphology, while Iba1-positive microglia were small with few processes. Overexpression of Iba1 was observed in SG (550.24 ± 51.20), TG (548.40 ± 69.90), and CanG (508.44 ± 57.94) groups, with no significant differences among them. The lowest densities were found in ConG (102.24 ± 4.35) and DG (115.83 ± 15.09) groups. Cannabixir® Medium Flos combined with donepezil or with donepezil–tramadol showed moderate to reduced Iba1 expression: CanDG decreased by 64.47% (p < 0.05) and CanDTG by 53.89% (p < 0.001) relative to CanG.

Casp-3 immunoreactivity in the hippocampus was highest in SG (658.02 ± 25.21), representing a 381.69% increase versus ConG (p < 0.001). Tramadol also significantly increased apoptosis (p < 0.05), whereas donepezil and Cannabixir® Medium Flos treatments reduced Casp-3 expression. The lowest Casp-3 levels were recorded in CanDG (63.57 ± 19.54), significantly lower than SG, TG and CanTG (p < 0.01–0.001) (Figure 7).

IL-6 was overexpressed in SG (679.92 ± 68.30), significantly higher than all other groups. CanDG group produced the lowest IL-6 expression (79.62 ± 48.41), reducing levels by 66.88% versus CanTG group (p < 0.05) and 88.29% versus SG group (p < 0.001) (Figure 7).

NeuN immunolabeling showed the highest expression in CanG (801.60 ± 54.09), significantly higher than ConG, SG, DG and TG groups (p < 0.01), with no significant difference from CanDG (695.73 ± 61.62). CanDTG and CanTG groups exhibited reduced to moderate NeuN expression (Figure 7).

Neuronal density in the dentate gyrus reflected these trends: CanG rats had the highest density (0.51 ± 0.08), significantly higher than all other groups (p < 0.01–0.001). The lowest densities were observed in SG (p = 0.001) and TG (p < 0.001) groups (Table 4).

## 4 Discussions

Chronic neuropathic pain often coexists with cognitive decline, worsening functional deficits and reducing quality of life in patients with dementia (Oh et al., 2024). Management of this comorbidity is challenging, as conventional analgesics, including opioids like tramadol, may pose additional risks (Musich et al., 2021). Preclinical evidence suggests that prolonged tramadol exposure can lead to hippocampal atrophy, impair learning and memory (Raj et al., 2019; Hussein et al., 2020), yet human studies remain limited and inconclusive (Taipale et al., 2018). Observational studies frequently overlook critical factors such as lifestyle, competing mortality risks and duration of opioid exposure (Oh et al., 2024), underscoring the need for alternative strategies that alleviate pain without compromising cognition.

To investigate potential therapeutic interventions in a clinically relevant context, we employed a dual *in vivo* model combining chronic neuropathic pain induced by sciatic nerve ligation with scopolamine-induced cognitive impairment. While scopolamine administration does not recapitulate hallmark pathological features of Alzheimer’s disease, such as amyloid-β deposition or tau hyperphosphorylation, it consistently produces deficits in learning, memory and synaptic plasticity, as well as associated neuroinflammatory responses (Stanciu et al., 2023; Miravalles et al., 2025). This pharmacological model has been extensively validated across multiple species, including rodents (mice and rats) and zebrafish, enabling cross-species evaluation of cognitive function, neurochemical alterations and pharmacological interventions (Déciga-Campos et al., 2025; Chen and Yeong, 2020; Budzynska et al., 2015). While limitations exist regarding the full translational relevance to human Alzheimer’s disease, this model remains a valuable tool for preclinical testing of interventions targeting cholinergic deficits, synaptic dysfunction and neuroinflammatory processes (Déciga-Campos et al., 2025; Miravalles et al., 2025). By combining neuropathic pain with cognitive impairment, this approach closely replicates the clinical scenario in which dementia is accompanied by persistent neuropathic pain, enabling simultaneous assessment of peripheral nociceptive processing and central cognitive deficits. The endocannabinoid system plays a central role in maintaining neuronal homeostasis, modulating pain signaling and limiting neuroinflammatory responses via CB_1_ and CB_2_ receptors (Simankowicz and Stępniewska, 2025). Cannabinoid-based compounds have therefore attracted attention as multi-target agents capable of providing both neuroprotective and anti-inflammatory effects (Kaur et al., 2016). Although the pharmacodynamics of isolated cannabinoids such as CBD and THC are well-characterized, standardized plant-based formulations like Cannabixir® Medium Flos have been less extensively studied. Our study aimed to bridge this gap by assessing the therapeutic potential of an EU-GMP certified Cannabis sativa L. strain in a complex pathological context.

Cannabixir® Medium Flos administration was well tolerated, with no significant changes in body weight or serum biochemical parameters, indicating systemic safety and metabolic stability. These observations are consistent with previous reports that cannabinoids, particularly THC and CBD, can modulate multiple signaling pathways without inducing hepatotoxicity or renal dysfunction, even under chronic exposure (Stanciu et al., 2024; Tudorancea et al., 2025). Furthermore, cannabinoids may exert hepatoprotective and renoprotective effects through CB_1_- and CB_2_-mediated antioxidant and anti-inflammatory mechanisms (Pan et al., 2009; Mecha et al., 2012; Carmona-Hidalgo et al., 2021). CB_1_ receptors, predominantly expressed in the brain but also present in hepatocytes and hepatic stellate cells, together with CB_2_ receptors, primarily localized in immune cells such as Kupffer and hepatic stellate cells, contribute to maintaining cellular homeostasis and limiting oxidative stress (Louvet et al., 2011; Mukhopadhyay et al., 2011). Mild alterations observed in the tramadol-treated group align with previously reported hepatocellular stress associated with opioid metabolism (Barbosa et al., 2020), supporting the hypothesis that Cannabixir® Medium Flos co-administration may mitigate systemic adverse effects under long-term treatment conditions.

Behavioral nociceptive testing using the Tail Flick, Hot Plate and Randall–Selitto assays revealed a marked and time-dependent analgesic effect of Cannabixir® Medium Flos. Obvious increases in response latencies were observed as early as 30 min post-administration, becoming highly significant at 120 min. Importantly, the CanDTG group exhibited prominently longer latencies than the tramadol-treated group, indicating a more potent and sustained analgesic action. These results strongly support the efficacy of Cannabixir® Medium Flos in modulating nociceptive responses, consistent with cannabinoid-mediated CB1 central inhibition and CB2 peripheral anti-inflammatory mechanisms (Smith and Martin, 1992; Starowicz and Finn, 2017). In contrast, mechanical sensitivity assessed via the Randall–Selitto test showed only limited changes following treatment. The significant decrease in mechanical thresholds at T0 compared to the post-operative baseline confirmed the successful induction of neuropathic pain, establishing a reference for mechanical hypersensitivity. Subsequent time points did not demonstrate further significant alterations, suggesting that cannabinoid compound primarily modulated thermal nociceptive responses rather than mechanical thresholds. This differential effect aligns with prior evidence that cannabinoids preferentially attenuate thermal and chemical nociception, while mechanical nociceptive pathways may be less sensitive to cannabinoid-mediated modulation (Starowicz and Finn, 2017; Bouchet and Ingram, 2020). Collectively, these results indicate that Cannabixir® Medium Flos exhibits stimulus-specific and time-dependent analgesic properties, particularly effective against thermal nociceptive stimuli. The enhanced analgesic effect observed in concurrent therapy, especially in the CanDTG group, further suggests potential synergistic interactions with donepezil and tramadol. However, these findings should be interpreted with caution, as the study was limited by sample size and the use of a single animal model. Further research is warranted to confirm these effects and to investigate their underlying mechanisms across different types and chronicity of pain, pain-processing regions represents an important limitation and that future studies will extend the investigation to cortico-striatal and limbic circuits.

Formal cognitive testing was not conducted to minimize animal stress, in accordance with the 3Rs principles (Replacement, Reduction, Refinement), particularly as multiple nociceptive assays were already included. The pharmacokinetic characteristics of this *Cannabis sativa* L. strain, previously described by our group (Filipiuc et al., 2023), support the absence of central psychoactive effects at the administered doses. Psychoactive THC remains undetectable in serum at oral doses up to 600 mg/kg and is largely replaced by its acidic precursor tetrahydrocannabinolic acid-A (THCA-A), which exhibits markedly reduced blood–brain barrier penetration and a CB1 receptor affinity at least 62-fold lower than THC. At the doses used in this study, THCA-A primarily mediates peripheral effects through transient receptor potential channels and cyclooxygenase 1/2 modulation, without activating central CB1 receptors. Importantly, the scopolamine-induced cognitive impairment model has been extensively validated by both our research team and the broader literature, demonstrating consistent deficits in hippocampal-dependent learning and memory, synaptic plasticity, and associated neuroinflammatory responses (Poceviciute et al., 2025; Stanciu et al., 2023; Bhuvanendran et al., 2018). These findings support its suitability as a preclinical tool for assessing cognitive impairment in the context of neuropathic pain.

The immunohistochemical analyses focused on the hippocampus, a brain region critically involved in learning, memory and synaptic plasticity, which are prominently affected in the scopolamine-induced cognitive impairment model (Miravalles et al., 2025). A panel of well-established markers was selected to comprehensively assess neuronal health and neuroinflammatory responses: NeuN for neuronal integrity, GFAP and Iba1 for astrocytic and microglial activation, Caspase-3 for apoptosis, and IL-6 and CD-163 for neuroinflammation. This targeted approach allowed the evaluation of key cellular processes relevant to cognitive function and neuropathic pain while maintaining a manageable scope for analysis. In the hippocampus, treatment with the cannabinoid compound, either alone or in combination with donepezil or tramadol, reduced GFAP and Iba1 expression, indicating attenuation of astrocytic and microglial activation. Notably, the combination of Cannabixir® Medium Flos with donepezil (CanDG group) produced an even greater reduction in GFAP expression, suggesting that the cannabinoid formulation may potentiate the anti-inflammatory effects of donepezil. These findings are consistent with previous studies showing that cannabinoids can suppress neuroinflammation by downregulating proinflammatory cytokines such as IL-6 and TNF-α (Puffenbarger et al., 2000; Nagarkatti et al., 2009). High astrocytic densities observed in the SG and TG groups reflect a pronounced neuroinflammatory response, in line with reports linking opioid exposure to oxidative stress and glial activation (Park et al., 2024; Abdelmoez, 2024). Several studies also support the role of GFAP upregulation in opioid dependence and tolerance (Torkzadeh-Mahani et al., 2019; Marie-Claire et al., 2004), where chronic drug-induced astrogliosis is considered an innate immune response to neurotoxicity and brain injury, potentially affecting synaptogenesis, neurogenesis, apoptosis and/or necrosis (Hussein et al., 2020). In our study, the increased GFAP expression aligns with signs of glial proliferation and hypertrophy.

Caspase-3 and IL-6 immunoreactivity patterns in the hippocampus indicate that scopolamine induces both apoptotic and inflammatory responses, consistent with its well-documented neurotoxic and cholinergic-blocking effects (Cheon et al., 2021). Tramadol produced a similar but less pronounced increase in Caspase-3 expression, aligning with reports of opioid-related oxidative stress and neuronal vulnerability (Abdelmoez, 2024). In contrast, treatment with donepezil or Cannabixir® Medium Flos markedly reduced apoptotic activity, with the greatest suppression observed in the combined Cannabixir®–donepezil group. This synergistic effect suggests that the cannabinoid formulation enhances donepezil’s neuroprotective efficacy, possibly through modulation of CB1/CB2 receptor-mediated pathways that inhibit caspase activation and promote neuronal survival. Likewise, the significant reduction in IL-6 levels following Cannabixir®–donepezil co-administration supports the anti-inflammatory potential of this therapy. Previous studies have shown that cannabinoids downregulate pro-inflammatory cytokines and microglial activation in models of neurodegeneration (Lisboa et al., 2016; Martín-Moreno et al., 2011), while donepezil exerts indirect anti-inflammatory effects through cholinergic enhancement (Cui et al., 2019). The nearly 90% suppression of IL-6 observed here is consistent with these findings and reinforces the hypothesis that concurrent targeting of cholinergic and endocannabinoid systems can effectively mitigate neuroinflammatory signaling.

In parallel, NeuN immunolabeling revealed preserved neuronal integrity in both Cannabixir® Medium Flos-treated and combined therapy groups, whereas the tramadol-treated group exhibited notable neuronal loss, though less severe than scopolamine alone. Comparatively, the CanDG and CanDTG groups displayed the highest neuronal densities, underscoring the superior neuroprotective effect of concurrent Cannabixir® Medium Flos and donepezil treatment. These findings highlight that, while tramadol may partially mitigate pain-related stress, it does not provide the same level of hippocampal protection as Cannabixir® Medium Flos or its combination with donepezil. This outcome corresponds with literature demonstrating that cannabinoids exert neuroprotective effects by limiting excitotoxicity, oxidative damage and apoptosis (Udovin et al., 2020), while donepezil promotes synaptic plasticity and neuronal resilience (Wu et al., 2024). Taken together, these results support a complementary mechanism through which Cannabixir® Medium Flos and donepezil act to preserve hippocampal structure and function by simultaneously suppressing inflammation and apoptosis.

The chronic ligation model produces profound alterations in the sciatic nerve, including demyelination, myelin sheath degeneration, infiltration of inflammatory cells and disorganization of nerve fibers (Al-Qudah and Al-Dwairi, 2016). In our study, these pathological changes were evident across all experimental groups, including animals treated with scopolamine, tramadol, donepezil, as well as those receiving cannabinoid alone or in combination. The extent of nerve damage varied according to the treatment regimen. The SG group exhibited the most severe impairments in axonal regeneration, characterized by marked Caspase-3 and IL-6 overexpression, moderate GFAP levels, comparatively low Iba-1 and CD163 immunoreactivity relative to controls. Conversely, the most favorable regenerative outcomes were consistently observed in the groups treated with cannabinoid compound. Their relative efficacy in promoting sciatic nerve recovery appeared to follow the order: CanDG > CanDTG > CanTG > CanG, consistent with Schwann cell activation and axonal regeneration. Tramadol treatment increased GFAP-positive cells. Similarly, Caspase-3 levels were lowest in these groups, highlighting the anti-apoptotic role of the cannabinoid compound. Inclusion of Cannabixir® Medium Flos, either alone or in combination with donepezil, tramadol or both, further increased Iba1-positive cell density (p < 0.01) compared with scopolamine or groups treated with donepezil or tramadol alone. While GFAP’s function in the central nervous system (CNS) is relatively well established, its role in the peripheral nervous system remains less defined. Some evidence suggests that GFAP expression in Schwann cells contributes to repair mechanisms (Jessen and Mirsky, 2019). Interestingly, GFAP-deficient mice exhibit normal CNS development, with nerve regeneration only modestly delayed compared to wild-type counterparts (Triolo et al., 2006; Berg et al., 2013). Although only a subset of brain structures and markers was examined, the observed modulation of these markers provides evidence supporting the neuroprotective and neuroreparative potential of Cannabixir® Medium Flos. Future studies could expand the analyses to additional brain regions and molecular targets to further elucidate the compound’s mechanisms of action.

## 5 Conclusion

The present study highlights the distinct therapeutic profiles of Cannabixir® Medium Flos in a dual model of chronic neuropathic pain accompanied by cognitive impairment. As a single agent, Cannabixir® Medium Flos demonstrated analgesic effects, attenuated neuroinflammatory responses, preserved hippocampal neuronal integrity and promoted peripheral nerve regeneration. Tramadol alone, while partially effective in alleviating pain, showed limited neuroprotective effects, highlighting its potential limitations in addressing the combined pathology of neuropathic pain and cognitive decline.

Combination therapies showed additive trends, with Cannabixir® Medium Flos together with donepezil (CanDG) or with donepezil and tramadol (CanDTG) exhibiting more pronounced effects on glial activation, apoptotic and pro-inflammatory markers, and neuronal and axonal integrity. These findings suggest that targeting cannabinoid-mediated, cholinergic and opioid-related mechanisms may provide a multi-faceted approach to managing pain and neurodegeneration, although further studies are needed to confirm these effects.

Overall, our results provide a preclinical basis for exploring combination strategies that integrate Cannabixir® Medium Flos with conventional agents. Future investigations should clarify the molecular pathways involved, including CB_1_/CB_2_ receptor signaling, modulation of neuroinflammation and Schwann cell-mediated peripheral nerve repair, as well as assess long-term safety and efficacy across diverse models of neurodegeneration and chronic pain. Such studies will be essential to determine the translational potential of these co-therapies and to guide the development of interventions that may improve functional outcomes and quality of life in patients with dementia and comorbid conditions.

## Data Availability

The raw data supporting the conclusions of this article will be made available by the authors, without undue reservation.
